# Association of Reduced Long-Lasting Insecticidal Net Efficacy and Pyrethroid Insecticide Resistance With Overexpression of *CYP6P4*, *CYP6P3,* and *CYP6Z1* in Populations of *Anopheles coluzzii* From Southeast Côte d’Ivoire

**DOI:** 10.1093/infdis/jiaa699

**Published:** 2020-11-11

**Authors:** Anne Meiwald, Emma Clark, Mojca Kristan, Constant Edi, Claire L Jeffries, Bethanie Pelloquin, Seth R Irish, Thomas Walker, Louisa A Messenger

**Affiliations:** 1 Department of Disease Control, Faculty of Infectious Tropical Diseases, London School of Hygiene and Tropical Medicine, London, United Kingdom; 2 Centre Suisse de Recherches Scientifiques en Côte d’Ivoire, Abidjan, Côte d’Ivoire; 3 US President’s Malaria Initiative and Entomology Branch, Division of Parasitic Diseases and Malaria, Center for Global Health, Centers for Disease Control and Prevention, Atlanta, Georgia, USA

**Keywords:** *Anopheles coluzzii*, insecticide resistance, *Plasmodium falciparum*, long-lasting insecticidal nets, Côte d’Ivoire, PBO, chlorfenapyr, clothianidin, *CYP6P4*, *CYP6P3*, *CYP6Z1*

## Abstract

**Background:**

Resistance to major public health insecticides in Côte d’Ivoire has intensified and now threatens the long-term effectiveness of malaria vector control interventions.

**Methods:**

This study evaluated the bioefficacy of conventional and next-generation long-lasting insecticidal nets (LLINs), determined resistance profiles, and characterized molecular and metabolic mechanisms in wild *Anopheles coluzzii* from Southeast Côte d’Ivoire in 2019.

**Results:**

Phenotypic resistance was intense: >25% of mosquitoes survived exposure to 10 times the doses of pyrethroids required to kill susceptible populations. Similarly, the 24-hour mortality rate with deltamethrin-only LLINs was very low and not significantly different from that with an untreated net. Sublethal pyrethroid exposure did not induce significant delayed vector mortality effects 72 hours later. In contrast, LLINs containing the synergist piperonyl butoxide, or new insecticides clothianidin and chlorfenapyr, were highly toxic to *A. coluzzii*. Pyrethroid-susceptible *A. coluzzii* were significantly more likely to be infected with malaria, compared with those that survived insecticidal exposure. Pyrethroid resistance was associated with significant overexpression of *CYP6P4*, *CYP6P3*, and *CYP6Z1*.

**Conclusions:**

Study findings raise concerns regarding the operational failure of standard LLINs and support the urgent deployment of vector control interventions incorporating piperonyl butoxide, chlorfenapyr, or clothianidin in areas of high resistance intensity in Côte d’Ivoire.

## Teaser

Intense insecticide resistance is compromising malaria vector control in Côte d’Ivoire, driven principally by metabolic resistance mechanisms. Study findings support the urgent deployment of interventions incorporating newly approved insecticides and synergists to interrupt malaria transmission by insecticide-resistant vector populations.

In Côte d’Ivoire, malaria is a serious public health problem with the entire population of about 26.2 million people at risk, and disease prevalence reaching as high as 63% in the southwest region [1]. Control of *Anopheles gambiae* sensu lato (s.l.), the major malaria vector species group, has been through the efforts of the National Malaria Control Programme, which has distributed insecticide-treated nets as the primary vector control intervention. Indoor residual spraying and larviciding in high transmission areas have been recommended as complementary strategies; implementation of the former commenced in late 2020 [2]. Estimates of net coverage across the country remain low, with the proportion of households with at least ≥1 insecticide-treated net per 2 persons rising from 31% in 2012 to 47% in 2016, and insecticide-treated net use stagnating at 40% of households reporting sleeping under a net the previous night in both survey years [2]. The most recent universal net campaigns in Côte d’Ivoire in 2017–2018 issued conventional, pyrethroid (deltamethrin) long-lasting insecticidal nets (LLINs), aiming to achieve 90% coverage and 80% use [2]. However, country-wide, multiclass insecticide resistance among populations of *A. gambiae* s.l. is a growing cause for concern because of potential operational failure of current vector control strategies, both locally and across the sub-Saharan region [2, 3].

Resistance to pyrethroid and carbamate insecticides in *Anopheles* mosquitoes was first reported from the central region of Côte d’Ivoire in the early 1990s [4–7]. Local resistance to the major insecticide classes recommended by the World Health Organization (WHO) for adult mosquito control—pyrethroids, carbamates, organophosphates, and organochlorines—evolved rapidly [8–10] and has been increasing in intensity, driven largely by selective pressures imposed by contemporaneous scale-up of public health vector control interventions (including those targeting malaria, trypanosomiasis, and onchocerciasis vectors) and use of agricultural pesticides [7, 11–14]. This escalation in resistance has now begun to compromise the insecticidal efficacy and community-wide impact of conventional, pyrethroid LLINs in Côte d’Ivoire [14, 15], although some levels of personal protection may still remain [15–17].

Among vector populations across Côte d’Ivoire, the L1014F *kdr* mutation is pervasive and has been implicated in some longitudinal trends in decreasing DDT and pyrethroid susceptibility [7, 11]; L1014S *kdr* and N1575Y resistance mutations have also been detected but at much lower frequencies [18]. Extreme carbamate (bendiocarb) resistance and pyrethroid cross-resistance in some *A. gambiae* sensu stricto (s.s.) populations are mediated by overexpression of *CYP6P3* and *CYP6M2* and duplication of the G119S *Ace-1* mutation [19]. To support and safeguard future malaria control efforts in Côte d’Ivoire, the current study evaluated the efficacy of conventional and next-generation LLINs for prospective distribution, determined current insecticide resistance profiles of *A. gambiae* s.l. (principally *Anopheles coluzzii*), and characterized underlying molecular and metabolic resistance mechanisms.

## METHODS

### Study Area and Mosquito Collections

The study protocol was approved by the Comité National d’Ethique des Sciences de la Vie et de la Santé (no. 069-19/MSHP/CNESVS-kp) and the London School of Hygiene and Tropical Medicine (nos. 16782 and 16899). Study activities were conducted in the village of Aboudé, rural Agboville, Agnéby-Tiassa region, southeast Côte d’Ivoire (5^°^55’N, 4^°^13’W), selected because of its high mosquito densities and malaria prevalence [1]. Adult mosquitoes were collected using human landing catches, inside and outside households from 6 pm to 6 am, for a total of 190 person/trap/nights between 5 and 26 July 2019. Unfed mosquitoes, morphologically identified as *A. gambiae* s.l. [20], were tested in bioassays that same day, after a brief recovery period; blood-fed mosquitoes were first held for 2–3 days to allow for blood-meal digestion.

### WHO Cone Bioassay Testing

Two types of LLIN were evaluated in this study. PermaNet 2.0 is a conventional LLIN treated with deltamethrin only (1.4 g/kg ± 25%) and PermaNet 3.0 is a piperonyl butoxide (PBO) synergist LLIN, consisting of a roof containing PBO (25g/kg) and deltamethrin (4 g/kg ± 25%) and side panels containing deltamethrin only (2.8 g/kg ± 25%). WHO cone bioassays were used to test the susceptibility of *A. gambiae* s.l. exposed to unwashed PermaNet 2.0, PermaNet 3.0 roof panels, and PermaNet 3.0 side panels [21]. To control for potential variation in insecticide/synergist content, each of 5 LLINs per type was cut into 19 pieces, measuring 30 × 30 cm, with each piece tested a maximum of 3 times.

### Resistance Intensity and Synergist Bioassay Testing

Centers for Disease Control and Prevention (CDC) resistance intensity bioassays were performed for 6 public health insecticides (pyrethroids: alpha-cypermethrin, deltamethrin, and permethrin; carbamate: bendiocarb; neonicotinoid: clothianidin; and pyrrole: chlorfenapyr) [22, 23]. The diagnostic doses of all insecticides were evaluated (including clothianidin [90 µg per bottle] [23] and chlorfenapyr [100 µg per bottle]) and 2, 5, and 10 times the diagnostic dose of pyrethroid insecticides were also used. Per test, knockdown was recorded at 15-minute intervals for 30 minutes (pyrethroids and bendiocarb) or 60 minutes (clothianidin and chlorfenapyr) of insecticide exposure. One-hour PBO preexposures were performed using WHO tube assays [24], before deltamethrin CDC bottle bioassay testing [22].

WHO cone and CDC resistance intensity bioassay data were interpreted according to the WHO criteria [21, 22]. Mosquitoes that died after exposure to a LLIN or 1× insecticide dose were stored at −20°C in RNAlater (Thermo Fisher Scientific) and were considered “susceptible” for genotypic analysis. Surviving mosquitoes were held and scored for mortality rate after 24, 48 and 72 hours to observe delayed mortality effects. Kaplan-Meier curves were used to visualize survival data, and Cox regression was used to compare postexposure survival. Immediate mortality rates after LLIN (60 minutes and 24 hours) or insecticidal exposure (30 or 60 minutes, depending on insecticide) were excluded. Surviving mosquitoes at 72 hours were stored at −20°C in RNAlater and were considered “resistant” for genotypic analysis.

### Mosquito Processing, Identification of *A. gambiae* s.l. Species Complex Members, and *Plasmodium falciparum* Detection

A subsample of field-caught mosquitoes tested in bioassays was selected for molecular analysis (n = 912). Approximately equal numbers of specimens were chosen to represent phenotypically “susceptible” or “resistant” mosquitoes for each LLIN type or insecticide dose, selected across different replicates and testing days to capture as much population-level variation as possible. RNA was extracted from individual whole-body mosquitoes according to standard protocols [23]. Field *A. gambiae* s.l. were identified to species level [25] and were screened for the presence of *Plasmodium falciparum* [26].

### Characterization of Insecticide Resistance Mechanisms: Target-Site Mutations

The same cohort of field mosquitoes (n = 912) was tested for the presence of L1014F *kdr* [27] and N1575Y mutations [28]. A subsample of mosquitoes (n = 49) that were exposed to bendiocarb, clothianidin or chlorfenapyr was tested for the presence of the G119S *Ace-1* mutation [29]. Pearson χ ^2^ and Fisher exact tests (when sample sizes were small) were used to investigate the statistical association between resistance status, allele frequencies, and deviations from Hardy-Weinberg equilibrium.

### Characterization of Insecticide Resistance Mechanisms: Metabolic Gene Expression

Relative expression of 5 metabolic genes (*CYP6P3, CYP6P4, CYP6Z1, CYP6P1,* and *GSTE2*) was measured in all field collected mosquitoes (n = 912), using multiplex quantitative real-time polymerase chain reaction (PCR) assays, relative to the housekeeping gene ribosomal protein S7 (*RPS7*) [30]. In addition, gene expression levels were measured in susceptible *A. coluzzii* N’gousso colony mosquitoes (n = 48). All samples were run in technical triplicate. Expression level and fold change of each target gene between resistant and susceptible field samples, relative to the susceptible laboratory strain, were calculated using the 2− ^ΔΔCT^ method incorporating PCR efficiency, normalized relative to the endogenous control gene (*RPS7*).

## RESULTS

### Mosquito Collections and Species Identification

A total of 4917 female *A. gambiae* s.l. mosquitoes were collected in Agboville, Côte d’Ivoire. Of those, 912, which were previously tested in either LLIN bioefficacy (n = 384) or resistance intensity (n = 528) bioassays, were selected for molecular species identification. Of the 912 selected, 805 (88.3%) were determined to be *A. coluzzii,* 75 (8.2%) were *A. gambiae* s.s., and 22 (2.4%) were *A. gambiae*–*A. coluzzii* hybrids; 10 individuals did not amplify.

### LLIN Efficacy

**Figure 1.**
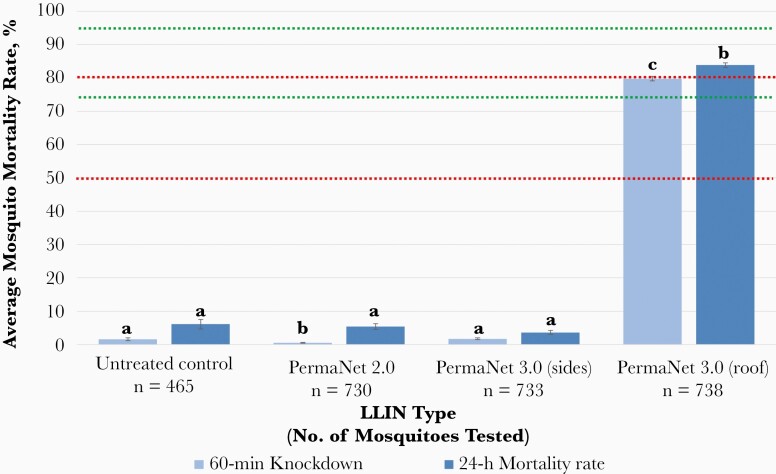
Bioefficacy of different unwashed long-lasting insecticidal nets (LLINs) against field-caught *Anopheles gambiae* sensu lato. Mean knockdown and mortality rates are shown with 95% confidence intervals, at 60 minutes and 24 hours, respectively, after 3-minute exposure to PermaNet 2.0 (deltamethrin only), side panels of PermaNet 3.0 (deltamethrin only), roof panels of PermaNet 3.0 (piperonyl butoxide plus deltamethrin), and an untreated control net. Knockdown or mortality rates in the same time period for each treatment sharing a letter do not differ significantly (*P* > .05). Green lines at ≥75% and ≥95% knockdown represent minimal and optimal effectiveness, respectively, at 60 minutes. Red lines at ≥50% and ≥80% mortality represent minimal and optimal LLIN effectiveness at 24 hours, respectively, as defined by the World Health Organization [21].

A total of 2666 field-caught *A. gambiae* s.l. were used to assess the bioefficacy of conventional pyrethroid-treated LLINs (PermaNet 2.0 and PermaNet 3.0 side panels) and next-generation synergist LLINs (PermaNet 3.0 roof panels), compared with an untreated control (Figure 1). Overall, *A. gambiae* s.l. knockdown and mortality rates with deltamethrin LLINs were very low and largely equivalent to those for the untreated control net (Figure 1). At 60 minutes, average mosquito knockdown rates with the untreated control, PermaNet 2.0, and PermaNet 3.0 side panels were 1.56% (95% confidence interval [CI], 1.13%–1.99%), 0.54% (.42%–.65%), and 1.75% (1.49%–2.0%), respectively. By contrast, average mosquito knockdown rates for PBO-containing PermaNet 3.0 roof panels were significantly higher (79.8% [95% CI, 79.07%–80.48%]; χ2 = 705.51, 968.65, and 937.33 [*P* < .001] vs untreated control, PermaNet 2.0, and PermaNet 3.0 side panels, respectively) (Figure 1).

At 24 hours, mortality rates with the untreated control, PermaNet 2.0, and PermaNet 3.0 side panels remained low (6.11% [95% CI, 4.71%–7.51%], 5.44% [4.58%–6.29%], and 3.66% [3.12%–4.19%], respectively), while those with PermaNet 3.0 roof panels increased only marginally but still remained significantly higher (83.81% [95% CI, 83.15%–84.47%]; χ2 = 727.96, 914.61, and 963.09 [*P* < .001 for all] vs untreated control, PermaNet 2.0, and PermaNet 3.0 side panels, respectively) (Figure 1). PermaNet 3.0 roof panels reached minimal effectiveness (knockdown, ≥75%) 60 minutes after exposure and optimal effectiveness (mortality rate, ≥80%) at 24 hours. Neither of the deltamethrin-only LLINs reached either effectiveness threshold at any time point.

### Insecticide Resistance Intensity

**Figure 2.**
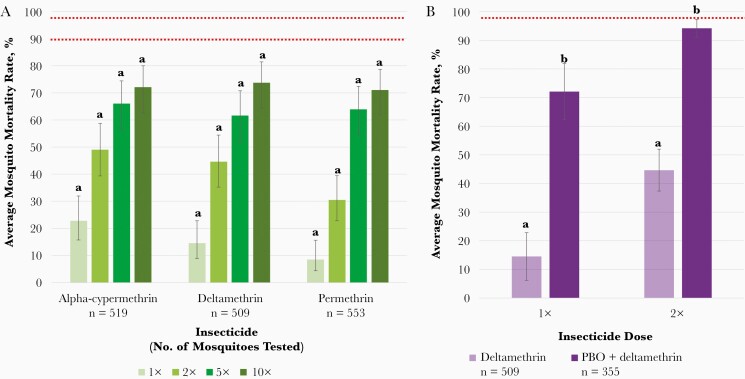
*A,* Resistance intensity of field-caught *Anopheles gambiae* sensu lato (s.l.) after exposure to 1, 2, 5, or 10 times the diagnostic dose of pyrethroid insecticides. Mean knockdown/acute toxicity rates after 30-minute exposure are shown with 95% confidence intervals (CIs). Knockdown or mortality rates at the same dose per insecticide sharing a letter do not differ significantly (*P* > .05). Mortality rates <90% (*lower red line*) represent confirmed resistance at the diagnostic dose (1×), and rates <98% (*upper red line*) indicate moderate to high-intensity resistance or high-intensity resistance at 5× and 10×, respectively, as defined by the World Health Organization [24]. *B,* Restoration of deltamethrin susceptibility of field-caught *A. gambiae* s.l. after preexposure to piperonyl butoxide (PBO). Mean knockdown/acute toxicity after 30-minute exposure to 1 or 2 times the diagnostic dose of deltamethrin with 95% CIs. Knockdown/mortality rates do not differ significantly between pyrethroid only and synergist plus pyrethroid sharing a letter (*P* > .05). Red line at 98% mortality rate represents metabolic resistance mechanisms partially involved [24].

A total of 2251 field-caught *A. gambiae* s.l. were tested in resistance bioassays. Intense pyrethroid resistance was evident with more than 25% of mosquitoes surviving exposure to 10 times the dose of insecticide required to kill a susceptible population (Figure 2A). At the diagnostic dose, mosquito mortality rates did not exceed 25% for any pyrethroid tested, which was consistent with the high survival rates observed during cone bioassays using conventional LLINs (Figure 1). In general, levels of resistance to alpha-cypermethrin, deltamethrin, and permethrin were not significantly different at each insecticide concentration tested (Figure 2A).

By comparison, carbamate tolerance was low, with a mean knockdown of 94.53% (95% CI, 92.11%–96.95%; n = 101) after 30 minutes of exposure to the diagnostic dose of bendiocarb. Similarly, high levels of susceptibility to new insecticides clothianidin and chlorfenapyr were observed, with mean mortality rates of 94.11% (95% CI, 93.43%–94.80%; n = 102) and 95.54% (94.71%–96.36%; n = 112), respectively, 72 hours after exposure to the tentative diagnostic doses. Preexposure to PBO increased the average *A. gambiae* s.l. mortality rate significantly, from 14.56% (95% CI, 6.24%–22.88%) to 72.73% (64.81%–79.43%) and from 44.66% (34.86%–54.46%) to 94.17% (91.12%–97.22%) after exposure to 1 or 2 times the diagnostic dose of deltamethrin (Figure 2B).

### Mosquito Survival After Insecticidal Exposure

**Figure 3.**
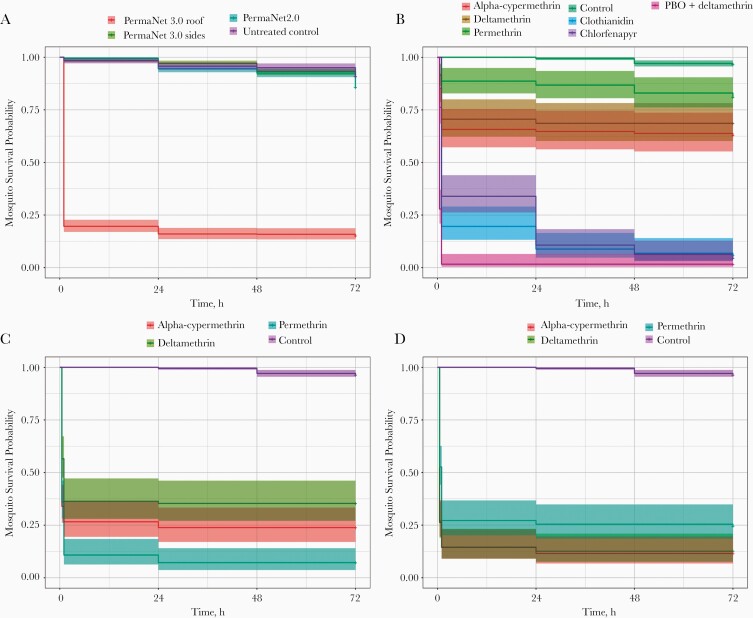
The longevity of field-caught *Anopheles gambiae* sensu lato after exposure to long-lasting insecticidal nets (LLINs) in World Health Organization cone assays (*A*) and 1× (*B*), 5× (*C*), and 10× (*D*) the diagnostic dose of pyrethroid insecticides in Centers for Disease Control and Prevention resistance intensity assays. Kaplan-Meier survival curves indicate the proportion alive each day after exposure. Immediate mortality rates after LLIN (60 minutes and 24 hours) or insecticidal exposure (30 or 60 minutes, depending on insecticide) were excluded.

All *A. gambiae* s.l. tested in LLIN bioefficacy or resistance intensity bioassays, were held for 72 hours, to assess any impact of insecticide or net exposure on delayed mortality rate. For LLIN bioassays, there was little evidence for any reduction in survival during this holding period (Cox regression *P* = .15, .27, and .85, respectively, for comparisons between untreated control and PermaNet 2.0, PermaNet 3.0 side panels, and PermaNet 3.0 roof panels) (Table 1 and Figure 3A). Exposure to the diagnostic doses of all insecticides in CDC bottle bioassays did not induce significant delayed mortality effects over 72 hours (Cox regression *P* > .05 for all insecticides compared with control, with the exception of chlorfenapyr [*P* = .02]) (Table 1 and Figure 3B). This phenomenon was also observed at increasing pyrethroid doses (Cox regression *P* > .05 for alpha-cypermethrin, deltamethrin, and permethrin 5× and 10× vs either the control or diagnostic dose) (Table 1; Figure 3C and 3D).

**Table 1.** Cox Proportional Hazard Model to Determine Impact of Long-Lasting Insecticidal Net/Insecticidal Exposure on Survival of Field-Caught *Anopheles gambiae* Sensu Lato 72 Hours After Exposure^a^

Insecticide Exposure	No. (No. of Events)	HRR (95% CI)	*P* Value^b^
Untreated netting		Reference	
PermaNet 2.0 (deltamethrin only)	1135 (1047)	1.095 (.968–1.239)	.15
PermaNet 3.0 side panels (deltamethrin only)	1157 (1088)	0.9664 (.9092–1.027)	.27
PermaNet 3.0 roof panels (PBO + deltamethrin)	563 (533)	1.007 (.939–1.079)	.85
Acetone control		Reference	
Alpha-cypermethrin 1×	676 (641)	1.006 (.9696–1.043)	.77
Deltamethrin 1×	683 (645)	0.9942 (.9539–1.036)	.78
Permethrin 1×	693 (661)	1.015 (.9698–1.062)	.52
Clothianidin 1×	698 (581)	1.208 (.9227–1.581)	.17
Chlorfenapyr 1×	708 (580)	1.692 (1.086–2.637)	.02
PBO + deltamethrin 1×	630 (577)	0.9662 (.2411–3.873)	.96
Alpha-cypermethrin 5×	633 (601)	0.9951 (.9407–1.053)	.86
Deltamethrin 5×	652 (610)	0.9942 (.9393–1.052)	.84
Permethrin 5×	636 (583)	0.9931 (.8638–1.142)	.92
Alpha-cypermethrin 10×	624 (587)	0.9951 (.917–1.08)	.91
Deltamethrin 10×	623 (588)	0.9943 (.9072–1.09)	.90
Permethrin 10×	656 (603)	1.026 (.9509–1.107)	.51
1× Insecticide dose		Reference	
Alpha-cypermethrin 5×	117 (92)	1.016 (.9069–1.138)	.78
Alpha-cypermethrin 10×	108 (78)	1.007 (.9403–1.078)	.84
Deltamethrin 5×	143 (105)	1.0 (.9035–1.107)	>.99
Deltamethrin 10×	114 (83)	1.0 (.9363–1.068)	>.99
Permethrin 5×	137 (94)	1.022 (.8528–1.225)	.81
Permethrin 10×	157 (114)	0.9952 (.9491–1.044)	.84

Abbreviations: CI, confidence interval; HRR, hazard rate ratio (ratio of hazard rate for control/reference group to hazard rate for treatment group; PBO, piperonyl butoxide.

^a^Immediate mortality rates after long-lasting insecticidal net (60 minutes and 24 hours) or insecticidal exposure (30 or 60 minutes, depending on insecticide) were excluded.

^b^Significance cutoff level defined as α = .05.

### Malaria Prevalence

Of the 912 *A. gambiae* s.l. mosquitoes assayed, 31 tested positive for *P. falciparum* (3.4%). For PCR-confirmed *A. coluzzii*, *P. falciparum* prevalence was 3.50% (28 of 805); the remaining 3 infections were in *A. gambiae* s.s. (4%; 3 of 75). By resistance phenotype, susceptible *A. coluzzii* (ie, those that died after pyrethroid exposure) were more likely to be infected with malaria, compared with resistant mosquitoes (χ2 = 4.6987; *P* = .03); infection rates were 5.94% (13 of 219) and 2.49% (10 of 401), respectively.

### Target-Site Resistance Mutations

L1014F *kdr* screening revealed that 92.2% (796/863) of *A. gambiae* s.l. mosquitoes harbored the mutation; 71.5% (617 of 863) were homozygous, 20.7% (179 of 863) were heterozygous, 5.1% (44 of 863) were wild type, and 2.7% (23 of 863) did not amplify. For PCR-confirmed *A. coluzzii*, L1014F *kdr* prevalence was 87.8% (707 of 805); 66.6% (536 of 805) were homozygous for the mutation, 21.2% (171 of 805) were heterozygous, 5.3% (43 of 805) were wild type, and 2.2% (18 of 805) did not amplify. For *A. coluzzii*, population-level L1014F *kdr* allele frequency was 0.83, with evidence for significant deviations from Hardy-Weinberg equilibrium (χ2 = 29.124; *P* < .001). There was no significant association between L1014F *kdr* frequency and the ability of *A. coluzzii*, to survive pyrethroid exposure, in either LLIN or resistance bioassays (χ2 = 2.0001 [*P* = .16] and χ2 = 3.6998 [*P* = .054], respectively). Similarly, there was no significant association between L1014F *kdr* and the ability of *A. coluzzii* to survive PBO preexposure and pyrethroid treatment, in either LLIN or resistance bioassays (χ2 = 0.0086; *P* = .93; Fisher exact test, *P* = .429, respectively).

For PCR-confirmed *A. gambiae* s.s., L1014F *kdr* prevalence was 95.3% (61 of 64); 89.1% (57 of 64) were homozygous for the mutation, 6.3% (4 of 64) were heterozygous, none were wild type, and 4.7% (3 of 64) did not amplify. There was no significant association between L1014F *kdr* frequency and ability of *A. gambiae* s.s. to survive pyrethroid or PBO preexposure and pyrethroid treatment (in either LLIN or resistance bioassays), because all tested individuals harbored this mutation (n = 61). For *A. gambiae* s.s., the population-level L1014F *kdr* allele frequency was 0.97, with no significant deviations from Hardy-Weinberg equilibrium (χ2 = 0.070; *P* = .79).

N1575Y screening revealed that 2.3% of *A. gambiae* s.l. mosquitoes (21 of 912) harbored the mutation; all were heterozygotes. N1575Y prevalence was 1.1% (9 of 805) and 16% (12 of 75) for PCR-confirmed *A. coluzzii* and *A. gambiae* s.s., respectively; 0.99% (9 of 912) did not amplify. There was no evidence for ongoing N1575Y selection in either species (χ2 = 0.026 [*P* = .87] and χ2 = 0.62 [*P* = .43] for *A. coluzzii* and *A. gambiae* s.s., respectively). For *A. coluzzii*, there was no significant association between N1575Y frequency and ability of mosquitoes to survive pyrethroid exposure, in LLIN or resistance bioassays (χ2 = 0.0001 [*P* = .99] and χ2 = 0.3244 [*P* = .57], respectively).

G119S *Ace-1* screening revealed that 55.1% of *A. gambiae* s.l. mosquitoes (27 of 49) harbored the mutation; all were heterozygotes. G119S *Ace-1* prevalence was 64.9% (24 of 37) and 27.3% (3 of 11) for PCR-confirmed *A. coluzzii* and *A. gambiae* s.s., respectively; 1 remaining *A. gambiae*–*A. coluzzii* hybrid was wild type. For *A. coluzzii*, population-level G119S *Ace-1* allele frequency was 0.32, with evidence of significant deviations from Hardy-Weinberg equilibrium (χ2 = 8.525; *P* = .004). For *A. gambiae* s.s., population-level G119S *Ace-1* allele frequency was 0.14, with no significant deviations from Hardy-Weinberg equilibrium (χ2 = 0.274; *P* = .60). For *A. coluzzii*, there was a significant association between G119S *Ace-1* frequency and surviving bendiocarb exposure (Fisher exact test, *P* = .005).

### Metabolic Resistance Mechanisms

**Figure 4.**
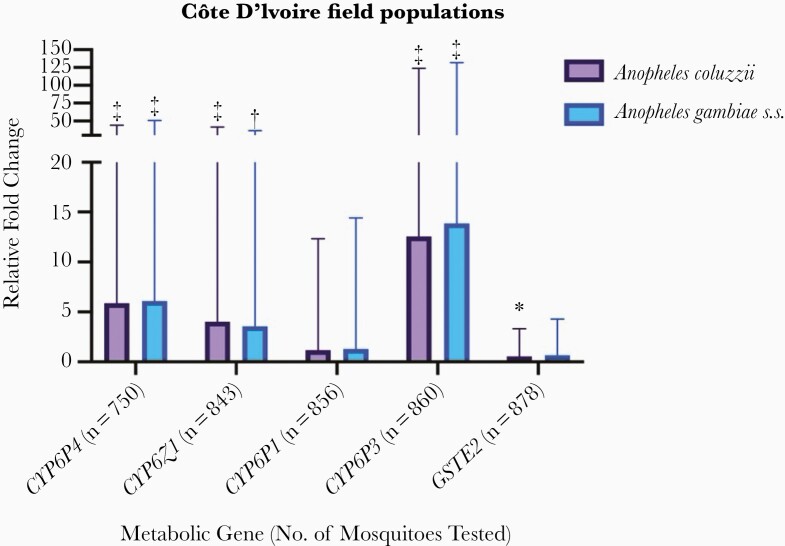
Metabolic gene expression in field *Anopheles coluzzii* and *Anopheles gambiae* sensu stricto (s.s.) populations relative to a susceptible colony population. Error bars represent 95% confidence intervals. Statistically significant differences in expression levels relative to the susceptible colony are indicated as follows: *P < .05; **P < .01; ***P ≤ .001.

**Figure 5.**
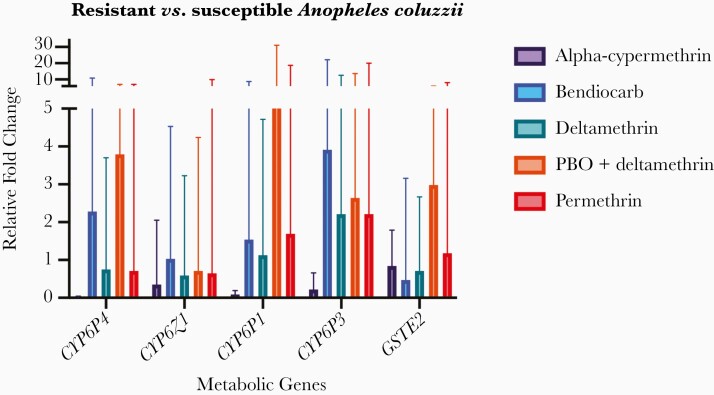
Metabolic gene expression in resistant versus susceptible field *Anopheles coluzzii*, which either died or survived after insecticidal exposure. Error bars represent 95% confidence intervals.

Comparison of metabolic gene expression levels in field populations of *A. coluzzii* and *A. gambiae* s.s. demonstrated significant up-regulation of *CYP6P4* (fold change, 5.88 [95% CI, 5.19–44.06] for *A. coluzzii* and 6.08 [5.43–50.64] for *A. gambiae* s.s.), *CYP6Z1* (4.04 [3.69–41.54] and 3.56 [3.24–36.25], respectively), and *CYP6P3* (12.56 [11.40–123.83] and 13.85 [12.53–132.03]), relative to a susceptible laboratory colony, respectively (Figure 4). More modest overexpression of *CYP6P1* and *GSTE2* was observed (*CYP6P1* fold changes, 1.18 [95% CI, 1.08–12.31] and 1.28 [1.17–14.40]; GSTE2, 0.56 [.48–3.32], and 0.67 [.58–4.29], for *A. coluzzii* and *A. gambiae* s.s., respectively) (Figure 4). The fold change levels did not differ significantly between the 2 species for any gene nor by malaria infection status in wild *A. coluzzii*. Comparison of metabolic gene expression in phenotyped field populations of *A. coluzzii* revealed lower fold changes overall, but notably, increased overexpression of *CYP6P3* in survivors of bendiocarb, deltamethrin, PBO plus deltamethrin, and permethrin (fold change, 3.91 [95% CI, 3.33–22.16], 2.21 [1.88–12.53], 2.64 [2.21–13.69], and 2.21 [1.99–20.03], respectively) (Figure 5).

## DISCUSSION

Côte d’Ivoire has hot spots with some of the highest levels of resistance of *Anopheles* mosquitoes to public health insecticides worldwide, with potentially severe implications for sustaining gains in malaria control [31]. To safeguard malaria vector control efforts and inform the design of effective resistance management strategies, involving tactical deployment of differing indoor residual spraying and LLIN modalities, there needs to be a clear understanding of contemporary phenotypic and genotypic insecticide resistance.

Our study detected intense pyrethroid resistance in southeast Côte d’Ivoire, as evidenced by high proportions of survivors, after exposure to 10 times the diagnostic doses of pyrethroids, as well as very low knockdown and 24-hour mortality rates for deltamethrin-only LLINs, equivalent to rates for an untreated net. These findings are largely in agreement with historical resistance profiles from this region [7, 10, 11] and indicate that conventional LLINs may no longer be operationally viable in areas of high pyrethroid resistance intensity. Previous phase II studies of pyrethroid-only LLINs in the central region of Côte d’Ivoire have demonstrated similarly poor efficacy with highly resistant *A. gambiae* s.l. populations but argued for the retention of some degree of personal protection [15–17].

Other observational cohorts have reported higher incidences of malaria among non–net users compared with users in areas of moderate to high pyrethroid resistance [17]. The extent of protective efficacy afforded by pyrethroid LLINs will likely reflect the strength of local vector resistance and levels of both net physical integrity and individual compliance [32, 33]; in Côte d’Ivoire, reported LLIN usage has been low, requiring additional behavioral interventions [2, 34]. Our findings of high mosquito mortality rates after exposure to clothianidin and chlorfenapyr and improved vector susceptibility with PBO treatment (on both LLINs and in resistance bioassays), are consistent with data from other sentinel sites across Côte d’Ivoire [16, 35, 36], and strongly support the deployment of vector control interventions incorporating these new active ingredients.

Study results indicate that *A. coluzzii* was the predominant local vector species during the rainy season, as observed previously [7], circulating sympatrically with smaller proportions of *A. gambiae* s.s. These 2 vector species commonly cohabit but can be genetically distinct in terms of resistance mechanisms [37, 38] and can also differ in larval ecology, behavior, migration, and estivation [39–41]. In general, resistance mechanisms in *A. coluzzii* are less well characterized, compared with *A. gambiae* s.s., in part because these vectors are morphologically indistinguishable and few studies present data disaggregated by PCR-confirmed species.

We observed several distinct features in our study, including, principally, evidence for ongoing selection of L1014F *kdr* and G119S *Ace-1* in *A. coluzzii*, which was absent in *A. gambiae* s.s. and higher proportions of N1575Y in *A. gambiae* s.s.; expression levels of metabolic genes were comparable between species. The lack of association between L1014F *kdr* genotype and mosquito phenotype, coupled with the identification of 3 CYP450 enzymes (*CYP6P4*, *CYP6P3,* and *CYP6Z1*) that were significantly overexpressed in field populations (some of which are known to metabolize pyrethroids and next-generation LLIN insecticides [42, 43]), indicate a key role for metabolic resistance in this *A. coluzzii* population. One notable difference in our data set, compared with previous findings in Agboville [7], was the finding of bendiocarb susceptibility. This may be attributable to small-scale spatial and longitudinal heterogeneity in resistance, which can be highly dynamic [37, 44], and/or phenotypic differences between vector species, complicating intervention choice for resistance management.

With the exception of chlorfenapyr, which is known to be a slow-acting insecticide, no delayed mortality effects were detected after insecticidal exposure; the format and dose used for clothianidin testing (another slow-acting insecticide [45]) were instead intended to measure acute toxicity within a 60-minute exposure period. Previous mathematical models using resistant mosquito colonies have suggested that sublethal insecticide treatment may still reduce vector lifespan and inhibit blood-feeding and host-seeking behaviors, thereby interrupting malaria transmission [46, 47]. Our observations are more compatible with reports from Burkina Faso, where different exposure regimens of wild, resistant *A. gambiae* s.l. populations to deltamethrin LLINs did not induce any delayed mortality effects [47]. Further assessment of sublethal effects are warranted across additional field populations with differing resistance mechanisms, to clarify the impact of insecticidal exposure on the vectorial capacity of resistant mosquitoes.

To date there is a paucity of data regarding the interactions between insecticide resistance and *Plasmodium* development [48]. In the current study, *A. coluzzii* that died after pyrethroid exposure were significantly more likely to be infected with malaria. This might be explained by elevated metabolic enzymes and/or prior pyrethroid exposure detrimentally affecting parasite development [49], although it is important to note that we did not detect any significant differences between gene overexpression in malaria-infected versus noninfected *A. coluzzii*. Alternatively, our sampled population may have been physiologically older, as phenotypic resistance is known to decline with age [50]. It is impossible to distinguish between these hypotheses using field-collected vector populations; the experimental design used in this study had other biological and technical limitations, which have been described in detail elsewhere [23, 37]

In conclusion, as new combination and bitreated vector control interventions become available for deployment, contemporary resistance information is crucial for the rationale design of management strategies and to mitigate further selection for particular resistance mechanisms. The results from the current study contribute to growing insecticide resistance data for Côte d’Ivoire, demonstrating a loss of bioefficacy of pyrethroid LLINs and supporting the use of new active ingredients (clothianidin, chlorfenapyr, and PBO). Study findings also highlight the need for expanded insecticide resistance surveillance, including monitoring of metabolic resistance mechanisms, in conjunction with studies to better characterize the impact of sublethal insecticide exposure on vectorial capacity and the interaction between insecticide resistance and *Plasmodium* parasite development.

## Notes

***Acknowledgments.*** The authors express their sincere thanks to M. Didier Dobri, Centre Suisse de Recherches Scientifiques en Côte d’Ivoire (CSRS) laboratory technician, and Fidele Assamoa for their support in mosquito collection and rearing, the chief and population of the village of Aboudé (Agboville), and the entomology fieldworkers of CSRS. They also thank Vestergaard for supplying the long-lasting insecticidal nets tested in this study.

***Author contributions.*** A. M., E. C., M. K., T. W., and L. A. M. designed the study. A. M., E. C., C. E., and B. P. led the entomology field activities and participated in data collection. A. M., E. C., C. L. J., T. W., and L. A. M. performed the molecular assays. A. M., E. C., M. K., C. E., C. L. J., B. P., S. R. I., T. W., and L. A. M. were responsible for data analysis and interpretation. L. A. M. drafted the manuscript, which was revised by all coauthors. All authors read and approved the final manuscript.

***Financial support***. This work was supported by the Sir Halley Stewart Trust (L.A.M.), the Wellcome Trust/Royal Society (http://www.wellcome.ac.uk and https://royalsociety.org; (101285/Z/13/Z to T.W.) and the US President’s Malaria Initative/Centers for Disease Control and Prevention (S.R.I.).

***Potential conflicts of interest.*** All authors: No reported conflicts. All authors have submitted the ICMJE Form for Disclosure of Potential Conflicts of Interest. Conflicts that the editors consider relevant to the content of the manuscript have been disclosed.
